# Flexural Testing of Steel-, GFRP-, BFRP-, and Hybrid Reinforced Beams

**DOI:** 10.3390/polym17152027

**Published:** 2025-07-25

**Authors:** Yazeed Elbawab, Youssef Elbawab, Zeina El Zoughby, Omar ElKadi, Mohamed AbouZeid, Ezzeldin Sayed-Ahmed

**Affiliations:** The American University in Cairo, New Cairo 11835, Egypt; yazeed.eysa@aucegypt.edu (Y.E.); youssef.eysa@aucegypt.edu (Y.E.); zeinaelzoghbi@aucegypt.edu (Z.E.Z.); o.elkadi@aucegypt.edu (O.E.); mnagiba@aucegypt.edu (M.A.)

**Keywords:** Basalt Fiber Reinforced Polymers (GFRP), hybrid-reinforced beams, ductility, flexure tests, Glass Fiber Reinforced Polymers (GFRP)

## Abstract

The construction industry is exploring alternatives to traditional steel reinforcement in concrete due to steel’s corrosion vulnerability. Glass Fiber Reinforced Polymer (GFRP) and Basalt Fiber Reinforced Polymer (BFRP), known for their high tensile strength and corrosion resistance, are viable options. This study evaluates the flexural performance of concrete beams reinforced with GFRP, BFRP, and hybrid systems combining these materials with steel, following ACI 440.1R-15 guidelines. Twelve beams were assessed under three-point bending to compare their flexural strength, ductility, and failure modes against steel reinforcement. The results indicate that GFRP and BFRP beams achieve 8% and 12% higher ultimate load capacities but 38% and 58% lower deflections at failure than steel, respectively. Hybrid reinforcements enhance both load capacity and deflection performance (7% to 17% higher load with 11% to 58% lower deflection). However, GFRP and BFRP beams show reduced energy absorption, suggesting that hybrid systems could better support critical applications like seismic and impact-prone structures by improving ductility and load handling. In addition, BFRP beams predominantly failed due to debonding and concrete crushing, while GFRP beams failed due to bar rupture, reflecting key differences in their flexural failure mechanisms.

## 1. Introduction

Fiber Reinforced Polymer (FRP) composites were initially developed for aerospace and military applications [1,2]; they soon found their way into civil engineering applications due to their superior corrosion resistance and high strength-to-weight ratio. In particular, Glass Fiber Reinforced Polymer (GFRP) and, recently, Basalt Fiber Reinforced Polymer (BFRP) have gained significant traction as concrete element reinforcement [3,4,5,6,7,8,9,10,11,12]. Compared to Carbon Fiber Reinforced Polymer (CFRP), GFRP and BFRP offer substantial advantages in cost, availability, and sustainability. CFRP exhibits higher tensile strength and lower elongation at break, but GFRP and BFRP are more economical and better suited for widespread infrastructure applications due to their natural (basalt) or synthetic (glass) sources [13].

FRP composites emerged as a solution to the limitations of steel reinforcement corrosion, particularly in environments where steel is prone to corrosion. However, a critical factor in the effectiveness of FRP-reinforced beams is the bond performance between FRP bars and concrete. Studies have shown that bond behavior, especially at the bar–concrete interface, significantly affects load transfer, cracking, and overall failure modes [14]. In general, FRP composites’ corrosion resistance, high tensile-strength-to-weight ratio, and durability in aggressive environments have made them an attractive choice in multiple structural applications. BFRP, a relatively newer material, offers similar advantages to GFRP, but with enhanced thermal stability and sustainability, and is increasingly being considered for fire-prone areas and sustainable building projects [15,16,17]. Unlike GFRP, BFRP fibers are derived from natural basalt rock, making them more environmentally friendly as their natural basalt composition reduces their carbon footprint. In general, the introduction of FRP materials into structural engineering applications, particularly in reinforcing concrete elements, allows for the development of structures with extended service life, reduced maintenance, and improved performance in aggressive environments [18,19,20,21,22].

GFRP bars typically exhibit tensile strength in the range of 500 to 1500 MPa, depending on the fiber content and the manufacturing process. However, GFRP’s elastic modulus is significantly lower than that of steel, leading to some concerns related to ductility. On the other hand, BFRP offers a higher elastic modulus and performs better in high-temperature environments, as it maintains its mechanical properties up to approximately 350 °C [15,20].

The design and application of FRP reinforcement are governed by several ACI guidelines [23,24,25]. These guidelines emphasize the importance of considering both strength and serviceability limits, particularly for flexural members where deflections and cracking need to be controlled. For example, GFRP-reinforced beams require careful attention to crack width limits, and design recommendations include the use of crack control reinforcement or increasing the section depth. This may constitute a drawback for using GFRP in reinforcing concrete elements. BFRP, while not yet fully integrated into design guidelines, is being studied for future inclusion in codes of practice and standards.

The durability of FRP-reinforced concrete structures is influenced by the bond between the FRP bars and the concrete [26,27]. Studies have shown that the bond strength between FRP bars and concrete is generally weaker than that of steel, which can lead to premature debonding failure if not properly accounted for during the design process [18,28]. To address this issue, ACI guidelines provide specific recommendations for the development length of FRP bars, which are typically longer than those required for steel reinforcement [23,24,25]. In addition to the bond strength concern, other factors, such as serviceability requirements, must be considered when assessing the performance of FRP-reinforced concrete beams. Few investigations have been performed on estimating the deflection of beams reinforced with GFRP and BFRP bars [29,30,31].

Despite the advantages of GFRP and BFRP in reinforced concrete beams, in addition to the above-mentioned constraints, other limitations still exist. One of the primary challenges is the higher upfront cost of FRP materials compared to traditional steel reinforcement. Although FRP offers long-term cost savings due to reduced maintenance and longer service life, the initial cost of materials can be prohibitive for some projects [32]. A lack of standardized design codes or guidelines for BFRP, as mentioned above, presents another challenge.

The future of GFRP and BFRP as structural concrete elements reinforcement looks promising as advancements in material science and sustainability continue to drive the development of new FRP technologies. Sustainability, a crucial factor nowadays, will shape the future of using FRP materials in structural engineering applications. BFRP, with its natural basalt composition, is well-positioned to meet the current growing demand for environmentally friendly structures. Advances in fiber production and composite manufacturing are expected to lower the upfront cost of FRP materials, making them more accessible for large-scale infrastructure projects [22].

While prior studies have explored the general behavior and failure patterns of FRP-reinforced beams, most have focused on either single-material systems or analytical modeling. Few have experimentally investigated comparative flexural behavior across GFRP, BFRP, and hybrid systems under consistent testing conditions. Research into hybrid reinforcement that combines FRP with steel offers significant potential for improving the overall performance of reinforced concrete structures [33,34,35,36,37]. These hybrid systems can provide the best of both worlds, combining the corrosion resistance of FRP with the high elastic modulus and ductility of steel, thereby overcoming some FRP limitations. The current investigation addresses that gap and evaluates the load-bearing capacity, ductility, and failure mechanisms using a comprehensive experimental and analytical approach.

### 1.1. Research Significance

This research underscores the transformative potential of Fiber Reinforced Polymers (FRPs) like GFRP and BFRP in modern construction, challenging traditional steel reinforcement in concrete beams. By incorporating advanced composites, this study not only aims to elevate structural durability and reduce environmental impact but also seeks to enhance the understanding of the flexural performance of FRP-reinforced beams. The findings could lead to broader adoption of FRP materials, influencing future design practices and FRP design guidelines, thereby contributing significantly to sustainability and innovation in structural engineering.

### 1.2. Research Objectives

As construction practices evolve and sustainability becomes a core focus, the use of FRPs in concrete beams has gained considerable attention. As mentioned earlier, these materials are considered alternatives to traditional steel reinforcement, offering advantages such as superior corrosion resistance, lightweight properties, and high tensile strength. However, despite these benefits, when used to reinforce beams, there is a critical need to conduct flexure testing (among other tests) to fully understand and compare the performance of GFRP, BFRP, and hybrid reinforcement systems under real conditions.

While GFRP and BFRP offer promising advantages over steel, there remain several unanswered questions regarding their flexural behavior in reinforced concrete beams. Key aspects such as flexural strength, deflection characteristics, ductility, cracking patterns, and failure modes need to be rigorously assessed and compared to those of traditional steel-reinforced beams. Furthermore, hybrid reinforcement systems, which combine steel and FRP, also require evaluation to determine whether they can provide enhanced performance in terms of load-bearing capacity and ductility.

The primary objective of this research is to experimentally investigate the flexural behavior of concrete beams reinforced with GFRP, BFRP, and hybrid combinations of these materials with steel. Specifically, this study aims to

- Compare the flexural strength of GFRP, BFRP, and hybrid reinforced beams to that of steel-reinforced beams;
- Analyze the deflection characteristics of each type of beam, focusing on the ductility behavior;
- Assess the cracking patterns and the overall failure modes to determine the suitability of GFRP, BFRP, and hybrid reinforcements for different structural applications;
- Examine whether hybrid reinforcement systems, such as Steel/GFRP, Steel/BFRP, and GFRP/BFRP combinations, offer better alternatives to traditional steel reinforcement, particularly in terms of ductility.

## 2. The Experimental Investigation: Materials and Methodology

While several studies have highlighted the advantages of FRPs, the actual performance of GFRP, BFRP, and hybrid reinforcements in terms of load capacity, cracking behavior, and failure mechanisms still needs further examination. In addition, ACI [23,24,25] provides guidelines for designing structural concrete reinforced with FRP bars, but further experimental validation is required to assess how these materials (particularly GFRP, BFRP, and hybrid reinforcement) behave in beams subject to flexure loading conditions.

**Figure 1:**
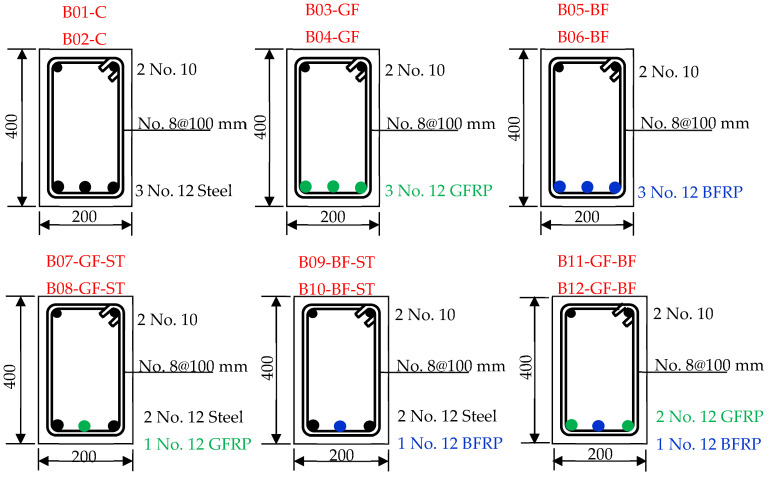
Cross-sections and reinforcement details of the tested beams.

Tests were conducted on 12 concrete beams (Table 1 and Figure 1), grouped into 6 sets:

1. Beams reinforced with steel (control group);
2. Beams reinforced with GFRP;
3. Beams reinforced with BFRP;
4. Beams reinforced with a hybrid of steel and GFRP;
5. Beams reinforced with a hybrid of steel and BFRP;
6. Beams reinforced with a hybrid of GFRP and BFRP.

It is worth mentioning that while GFRP and BFRP have almost identical mechanical properties, particularly in tensile strength and corrosion resistance, they differ in several key aspects, such as the modulus of elasticity, thermal resistance, cost, and sustainability. The hybrid GFRP–BFRP reinforcement is included in this study to explore potential synergistic behavior when combining these two materials, particularly in relation to failure modes and ductility. For instance, BFRP has a higher elastic modulus and better thermal performance, while GFRP is more established in structural codes and design practices. Investigating this hybrid configuration helps assess whether combining them could improve structural performance or mitigate limitations observed in single-material FRP systems.

The reinforcement configurations and cross-sectional dimensions presented in Table 1 are selected to reflect typical beam design practices in structural applications while maintaining a consistent geometry across all specimens to allow for direct comparison. Bar diameters and reinforcement layouts are chosen to ensure similar reinforcement ratios and to remain within the testing requirements of the laboratory setup. This approach enables a focused investigation into the effect of reinforcement type (steel, GFRP, BFRP, and hybrids) on flexural behavior under standardized conditions. It is worth mentioning here that the flexural steel and GFRP bars used in this investigation were deformed (ribbed), while the BFRP bars had a sand-coated surface finish. Both surface treatments were used to enhance the reinforcement bond with concrete.

In the current investigations, each set consists of two replica beams to increase the reliability of the test results. The flexure testing is intended to evaluate

- The flexural strength based on the nominal moment and load capacity;
- The deflection profiles to assess ductility;
- The crack propagation and failure modes of each reinforcement type;
- The ductility of beams compared to that of steel-reinforced beams.

This experimental study provides a comprehensive comparison of the flexural behavior of beams reinforced with GFRP, BFRP, and hybrid reinforcement systems. It identifies the advantages of hybrid systems in enhancing ductility and load-bearing capacity. Furthermore, it offers recommendations for incorporating GFRP and BFRP reinforcements into structural design codes, contributing to the development of sustainable and high-performance concrete structures.

The length of all the tested beams was 2.0 m, and they were tested over a span of 1.85 m. All the beams were reinforced for shearing with No. 8@100 mm and have top reinforcement with 2 No. 10. To isolate the effect of the bottom reinforcement material on flexural behavior, the top (compression) reinforcement and stirrups were kept as steel in all specimens. This ensured a consistent shear capacity and boundary condition across all tests, allowing for a focused comparison between steel, GFRP, BFRP, and hybrid systems in the tensile zone. The cross-section dimensions of the beams were 200 mm × 400 mm.

The concrete used in casting the beams has a mix designed to produce a 28-day cylinder strength *f_c_^/^* of 25 MPa. The mix is composed of

- A total of 350 kg/m^3^ Ordinary Portland Cement;
- A w/c ratio of about 0.4;
- A total of 615 kg/m^3^ fine aggregates (sand);
- A total of 1100 kg/m^3^ coarse aggregate (1″, 2‴ equal portions);
- Super plasticizer Skiament C494 Type F.

**Figure 2:**
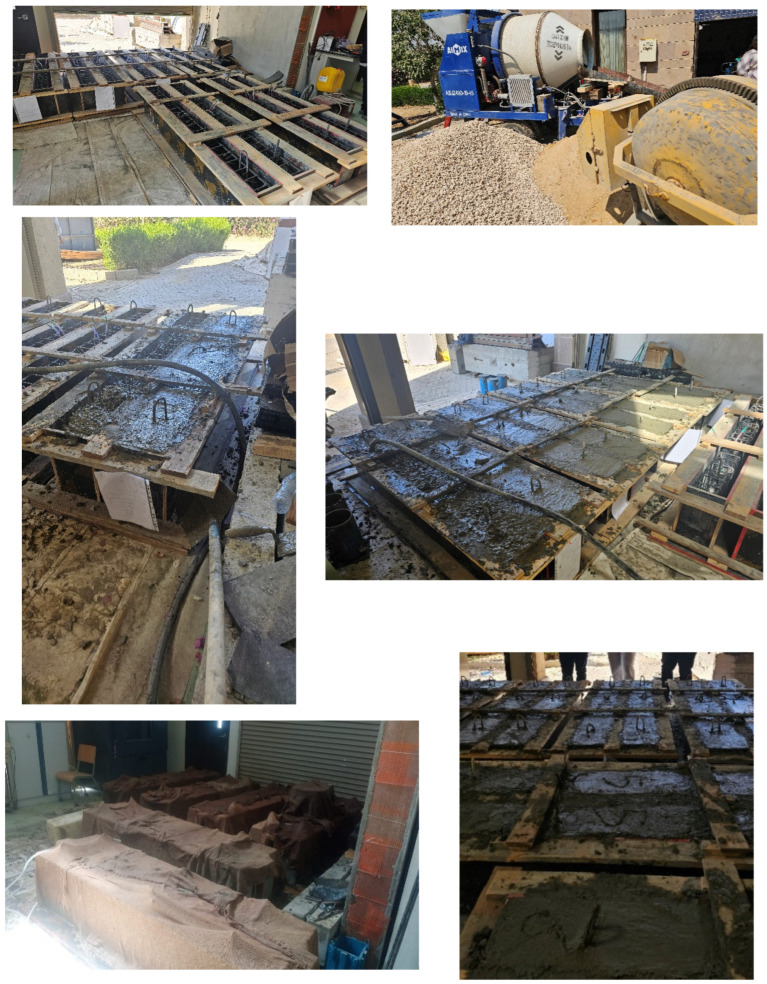
Casting and curing the beams.

A 1.0 m^3^ capacity mixer was used to mix the concrete, and 150 mm × 300 mm concrete cylinders were extracted from the same mix to test the 7d and 28d concrete compressive strength. Figure 2 shows the casting and curing of the casted beams.

**Figure 3:**
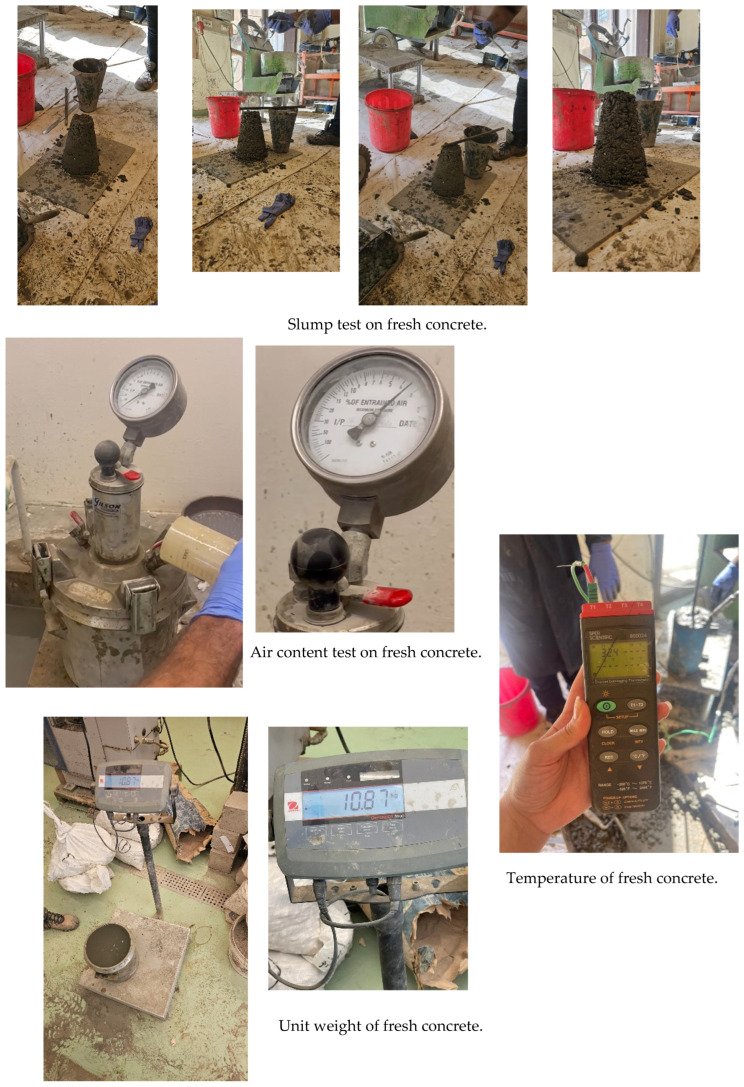
Tests performed on fresh concrete.

A concrete slump test was performed on the concrete mix, which yielded a 20 mm slump, indicating low concrete workability. The mix temperature measures 32.5 °C, which is about the recommended mix temperature (10–32 °C). The air content of the fresh concrete measures 3.2%. These tests are shown in Figure 3.

Three cylinders (150 mm × 300 mm) were tested after 7 days in compression, yielding a 7d concrete compressive strength of about 18 MPa. After 14 days, rebound tests using a Schmidt hammer were performed on the beams. Readings were taken on each beam, yielding an average cube (200 mm × 200 mm) strength of 24.5 MPa. Applying correction factors of 1.05 for the cube size and 1.25 for the cylinder strength yields *f_c_^/^*(14d) = 20.6 MPa. Three cylinders (150 mm × 300 mm) were tested after 28 days in compression, yielding a 28-day average concrete compressive strength of 24.8 MPa.

**Figure 4:**
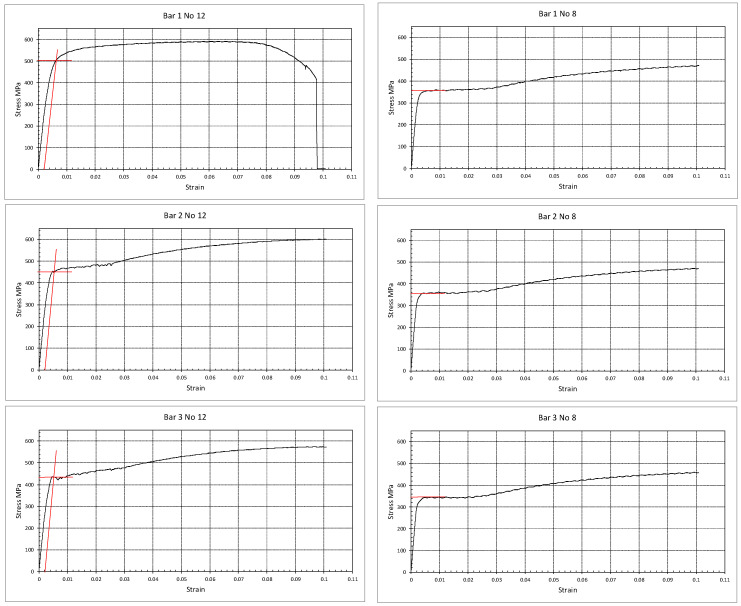
Uniaxial tension tests performed on the 12 mm (**left**) and 8 mm (**right**) bars used for flexure and shear reinforcement.

Six samples of the 12 mm and the 8 mm steel bars adopted for flexure and shear reinforcement were tested in uniaxial tension. The sample length was 400 mm with a gauge length of 260 mm. The results revealed a yield strength of 465 ± 35 MPa and 358 ± 2.9 MPa for the 12 mm bars and the 8 mm bars, respectively. Figure 4 shows the stress–strain relations resulting from the tests performed on these bars. The resulting values for the steel yield strength (465 MPa and 360 MPa) were adopted in the analytical calculations of the beams’ nominal moment and shear.

**Figure 5:**
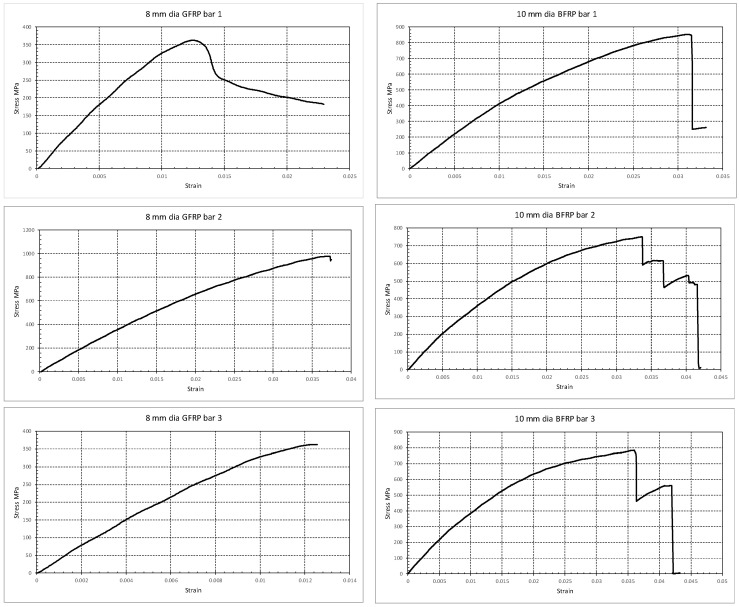
Uniaxial tension tests performed on the 8 mm GFRP bars (**left**) and 10 mm BFRP bars (**right**) used for flexure reinforcement.

The GFRP and BFRP bars were tested earlier [38], yielding a tensile strength of 1060 MPa and 1470 MPa, rupture strain of 0.022 and 0.026, and modulus of elasticity of 47.5 GPa and 59.7 GPa, respectively. Three samples of the GFRP bars (8 mm in diameter) were tested in uniaxial tension. Both ends of each bar were encased in metal sleeves and epoxy to minimize the gripping effect on the fibers. Despite these precautions, failure occurred in the gripping zones in all samples. Due to such premature failures during tensile testing, the resulting stress–strain data showed some variability. Therefore, the final reported values of elastic modulus and ultimate strength were determined by combining the reliable experimental results with reference values from ACI 440.6R-08 and relevant literature, ensuring consistency with published material behavior ranges. The stress–strain curves for the three tested samples are shown in Figure 5. The sample length is 400 mm with a gauge length of 260 mm. The results reveal a GFRP elastic modulus of 38.2 ± 1.4 GPa, an ultimate strength of 567 ± 292 MPa, and an ultimate strain of 0.0183 ± 0.008. These values are close to the values available in the literature, as listed above.

Three samples of the BFRP bars (10 mm in diameter) were tested in uniaxial tension. The sample length was 400 mm with a gauge length of 260 mm. Both ends of each BFRP bar were encased in metal sleeves and epoxy to minimize the gripping effect. Once again, failure occurred at the gripping zones; thus, test results were used in combination with previous tests listed above. The stress–strain curves for the three tested samples are shown in Figure 5. The results reveal a BFRP elastic modulus of 43.2 ± 0.6 GPa, an ultimate strength of 790 ± 41 MPa, and an ultimate strain of 0.033 ± 0.002.

**Figure 6:**
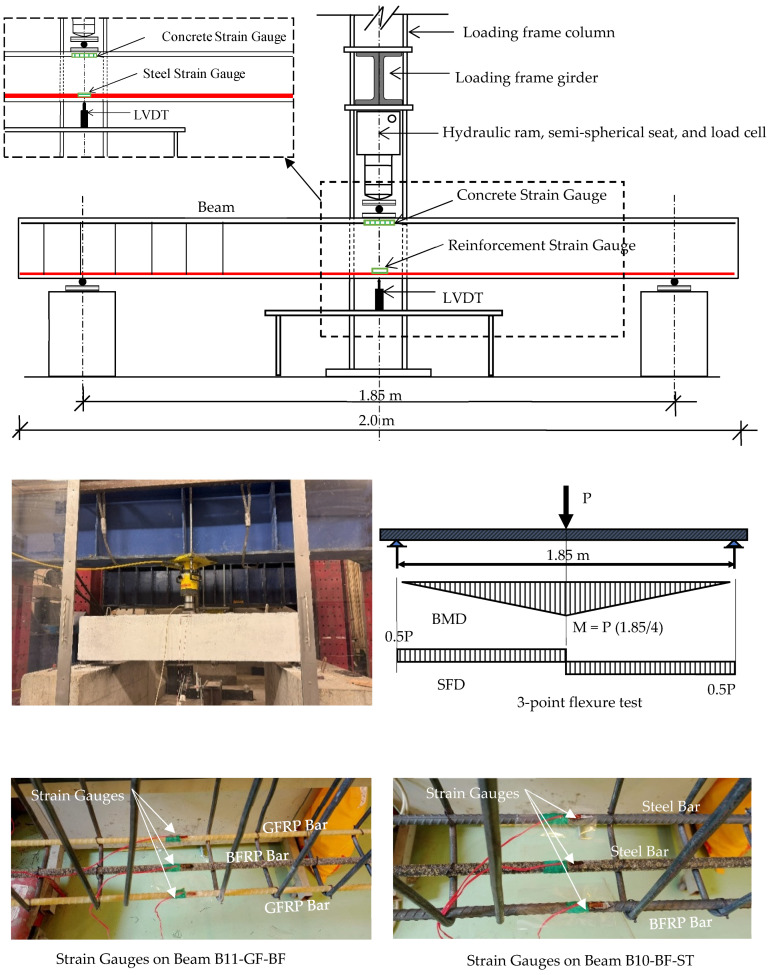
Test set-up and instrumentation.

All beams were tested in flexure in a three-point loading scheme monotonically to failure (Figure 6). Load was applied using an actuator connected to a load cell measuring the applied load. Mid-span deflection was measured using an LVDT (Linear Variable Differential Transformer). The LVDT was positioned vertically under the beam at mid-span, measuring the vertical deflection of the bottom surface (soffit) during loading. Three strain gauges were attached to the reinforcement (Figure 6) to measure the tensile strains in the reinforcement bars, while an additional strain gauge was attached to the concrete in compression. The concrete strain gauge was positioned at mid-span on the side face of the beam, aligned with the top compression fiber, to capture the compressive strain during flexural loading. All readings (load, deflection, and strains) were collected via a data acquisition system.

## 3. The Analytical Investigation

**Figure 7:**
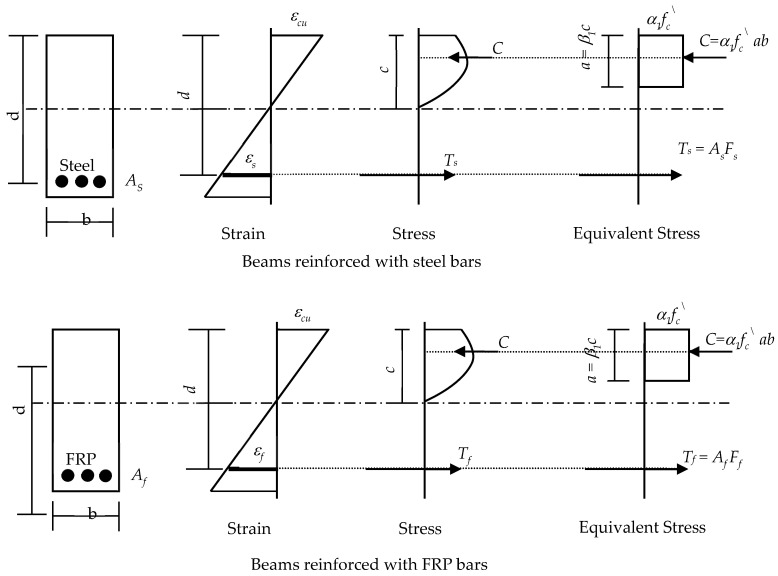
Distribution of stress and strains for nominal moment calculations.

An analytical investigation was conducted based on the ACI code of practice and guidelines [23,39] to compare the experimental results with the analytical predictions. Based on the equilibrium of forces and compatibility of strain conditions (Figure 7), the nominal moments for beams reinforced with steel, GFRP, BFRP, and hybrid reinforcement were calculated. ACI 318-19 [39] was used for beams reinforced with steel, while ACI 440.6R-08 [23] was adopted for beams reinforced with FRP and hybrid systems.

Furthermore, calculations for nominal shear were also performed based on the above-mentioned code of practice and guidelines for these beams. These calculations were included to ensure that all beams were adequately designed to fail in flexure rather than shear, in alignment with the study’s focus on flexural performance. In these calculations, the concrete, steel, and FRP mechanical properties listed in Table 2 were adopted. The mechanical properties of GFRP and BFRP bars used in the analytical calculations were based on values obtained from experimental tests and verified to fall within the standard ranges reported in ACI 440.6R-08. This approach ensures consistency while accounting for typical material variability.

### 3.1. Nominal Moment

With reference to Figure 7, the equilibrium of forces condition yields

(1) $$\begin{array}{l} \alpha_{1}\times f_{c}^{/}\times a\times b=A_{s}\times F_{s}+A_{f}\times F_{f}\\ F_{s}\leq F_{y-s}\;\;and\;\;F_{f}\leq F_{u-f} \end{array}$$

where *f_c_^/^* is the 28d concrete compressive strength; *F_s_* and *F_f_* are the stresses in the steel and FRP bars, respectively; *F_y-s_* is the yield stresses of the steel bars; *F_u-f_* is the rupture stress of the FRP bars; *A_s_* and *A_f_* are the areas of the steel and FRP bars, respectively; *a* is the depth of the equivalent concrete compression block; and *b* is the width of the beam’s cross-section.

Using the compatibility of the strain condition to check steel yielding and FRP rupture,

(2) $$\begin{array}{l} \frac{\varepsilon_{c}}{c}=\frac{\varepsilon_{s}}{d-c}\;\;;\;\;\frac{\varepsilon_{c}}{c}=\frac{\varepsilon_{f}}{d-c}\;\;;\;\;c=\frac{a}{\beta_{1}}\\ \varepsilon_{c}\leq \varepsilon_{cu}\;\;\varepsilon_{s}=\frac{F_{s}}{E_{s}}\leq \varepsilon_{y-s}\;\;\varepsilon_{f}=\frac{F_{f}}{E_{f}}\leq \varepsilon_{u-f} \end{array}$$

where *ε_c_*, *ε_s_*, and *ε_f_* are the strains in the concrete top fibers, steel bars, and FRP bars, respectively; *E_s_* and *E_f_* are the elastic moduli of the steel and FRP, respectively; *ε_y-s_* is the yield strain of the steel; *ε_u-f_* is the rupture strain of the FRP; *ε_cu_* is the concrete crushing strain; *d* is the depth of the reinforcement; and *c* is the depth of the neutral axis of the cross-section.

Solving Equations (1) and (2) simultaneously defines the failure mode and yields the nominal moment Mn, which is given by

(3) $$\begin{array}{l} M_{n}=A_{f}\times F_{f}\times \left(d-\frac{a}{2}\right)+A_{s}\times F_{s}\times \left(d-\frac{a}{2}\right)\\ P_{u-M}=\frac{4\times M_{n}}{L} \end{array}$$

where *P_u-M_* is the failure load corresponding to the three-point loading scheme (Figure 7), and *L* is the tested span of the beam (1.85 m).

### 3.2. Nominal Shear

The shear strength of the beam *V_n_* is calculated as follows:

(4) $$\begin{array}{l} V_{n}=V_{c}+V_{s}\\ V_{s}=\frac{A_{v}\times F_{y-v}\times d}{s}\\ \mathrm{For}\;\text{steel-reinforced}\;\mathrm{beams}:\;V_{c}=0.17\times \sqrt{f_{c}^{/}}\times b\times d\\ \mathrm{For}\;\mathrm{FRP}\;\mathrm{and}\;\text{hybrid-reinforced}\;\mathrm{beams}:\;V_{c}=\frac{2}{5}\times \sqrt{f_{c}^{/}}\times b\times \left(k\times d\right)\\ P_{u-V}=2\times V_{n} \end{array}$$

where *V_c_* is the concrete shear resistance, *A_v_* is the area of the stirrups, *F_y-v_* is the yield stress of the steel stirrups, *s* is the spacing between the stirrups, and *P_u-V_* is the failure load corresponding to the nominal shear. For FRP- and hybrid reinforced beams, the concrete shear strength is significantly reduced via a factor *κ* (ACI 440.1R-15) [24] since the contribution of the FRP to the dowel action was not determined. This factor is calculated based on the modular ratio between the FRP and the concrete *n_f_* and the FRP reinforcement ratio *ρ_f_* as follows:

(5) $$\begin{array}{c} k=\sqrt{2\rho_{f}n_{f}+{\left(\rho_{f}n_{f}\right)}^{2}}-\rho_{f}n_{f}\\ n_{f}=\frac{E_{f}}{E_{c}}\;\;and\;\;\rho_{f}=\frac{A_{f}}{bd} \end{array}$$

### 3.3. Cracking Moment

The beam’s cracking moment is calculated based on ACI 318-19 using the modulus of rupture *f_r_* as follows:

(6) $$\begin{array}{l} f_{r}=0.62\sqrt{f_{c}^{/}}\;\;\to M_{cr}=f_{r}\times S\\ P_{cr}=\frac{4\times M_{cr}}{L} \end{array}$$

where *S* is the elastic section modulus of the beam’s cross-section, *M_cr_* is the cracking moment, and *P_cr_* is the cracking load corresponding to the three-point loading scheme.

### 3.4. Results of the Analytical Investigation

Based on Equations (1) to (5), the analytically calculated nominal moment, nominal shear, and the expected failure modes for all beams are summarized in Table 3. Furthermore, using Equation (6) yields a cracking moment and cracking load of 16.5 kN·m and 35.7 kN, respectively.

## 4. Experimental Investigation Results and Discussion

The results of the experimental investigation are summarized in Table 4. The table lists the encountered failure loads, the deflection corresponding to the failure load, and the observed modes of failure for all beams.

### 4.1. Cracking Moment and Load–Deflection Relations

**Figure 8:**
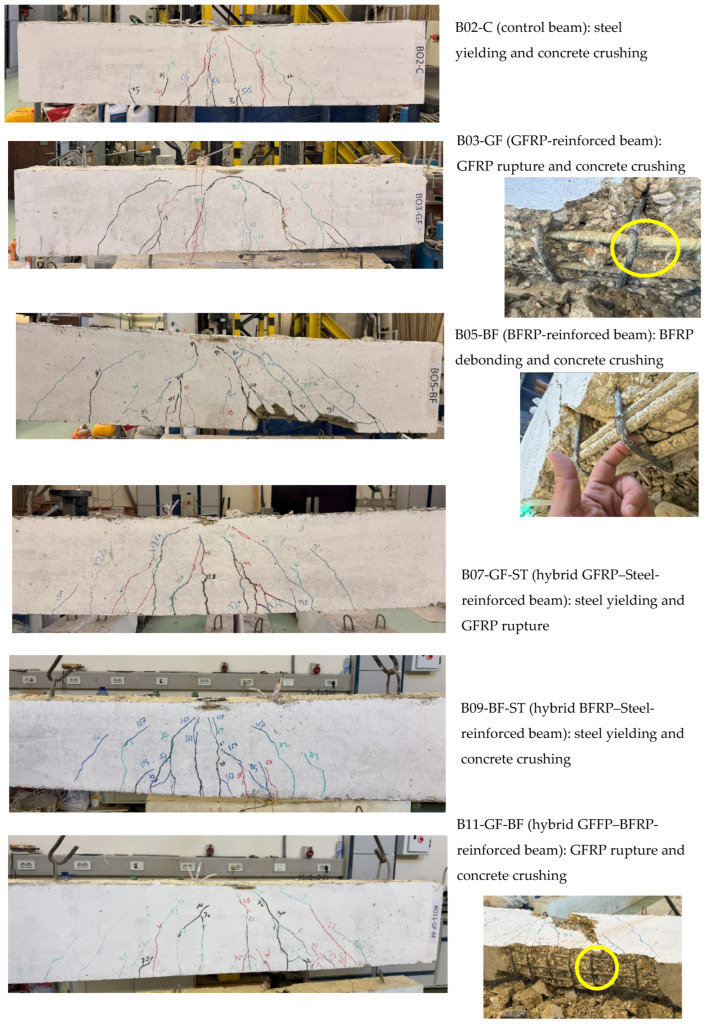
Cracking patterns and failure modes of the tested beams.

**Figure 9:**
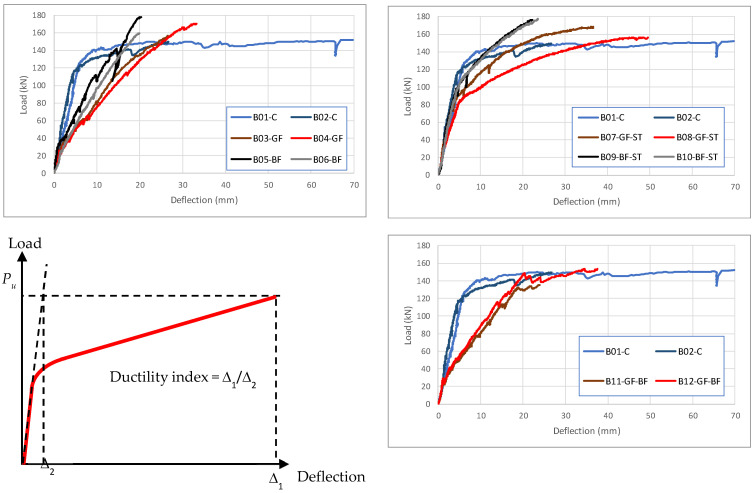
Load defection recorded for all tested beams and the adopted ductility index.

Cracking patterns and cracks’ propagation were recorded and marked during testing of the beams; these are shown in Figure 8. The load–deflection curves (Figure 9) and the cracking patterns show that all beams exhibit cracking around the values calculated using Equation (6). Cracks appeared and propagated in the vicinity of the beams’ midspan, indicating flexure failure for all beams. As such, as indicated earlier via the previous calculations for shear capacity, the experimental observations indicate that all beams failed in flexure.

Table 4 and Figure 8 also show the failure modes encountered experimentally. All failure modes agreed with those predicted analytically, except for beams reinforced with BFRP bars, where debonding of these bars was encountered before concrete crushing.

### 4.2. Ductility

To compare the ductility of the tested beams, a ductility index was calculated based on the concept shown in Figure 9. The areas under the load–deflection curves were calculated for all the tested beams to indicate the energy absorption capacity (toughness) of the beam. This area indicates the ability of the beam to sustain dynamic or cyclic loads. The said areas are calculated and listed in Table 5 for all beams. They are also correlated to the control beam traditionally reinforced with steel bars in this table. Furthermore, the table compares the ductility index of all the tested beams to the control beams traditionally reinforced with steel bars.

### 4.3. Failure Modes

**Figure 10:**
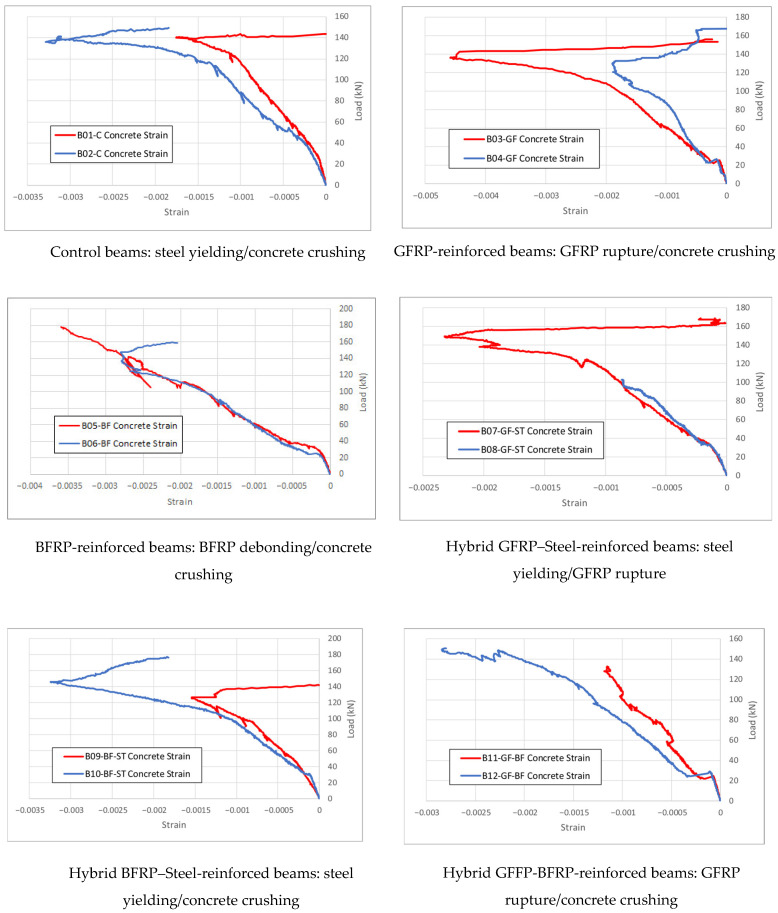
Concrete compression strain measurements for all the tested beams.

The failure modes observed during the experimental investigation are listed in Table 4 and shown in Figure 8. All failure modes agree with those analytically predicted except for beams reinforced with BFRP, where debonding of the BFRP from concrete was encountered before concrete crushing, as shown in Figure 4. The compressive concrete stains recorded during testing of all the beams are plotted in Figure 10.

Knowing that the crushing of concrete takes place at around 0.003 strain, the shown results also agree with the failure modes outlined above for the beams.

**Figure 11:**
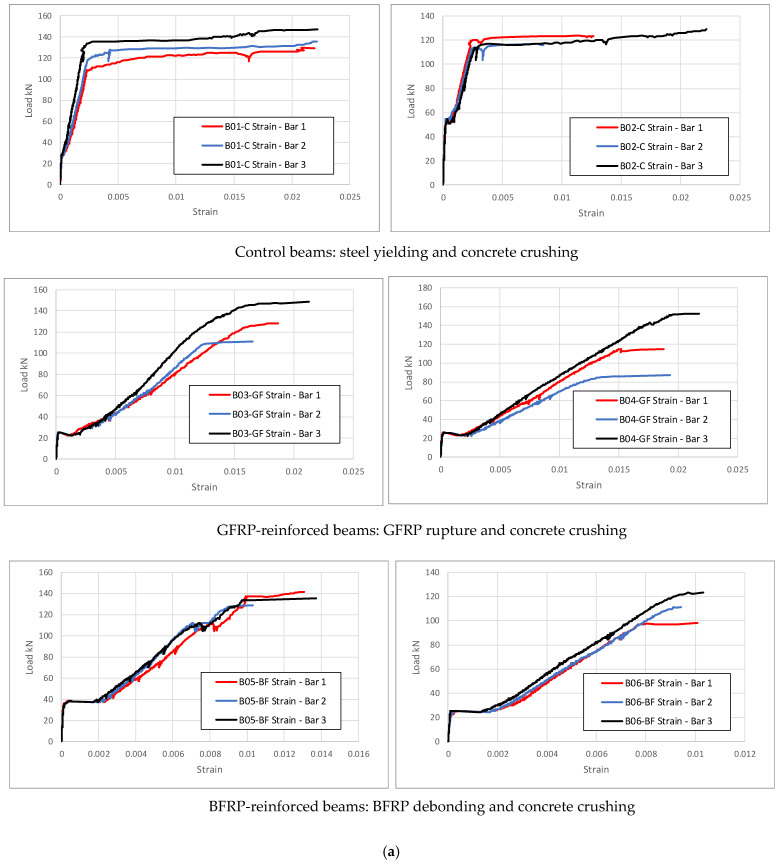

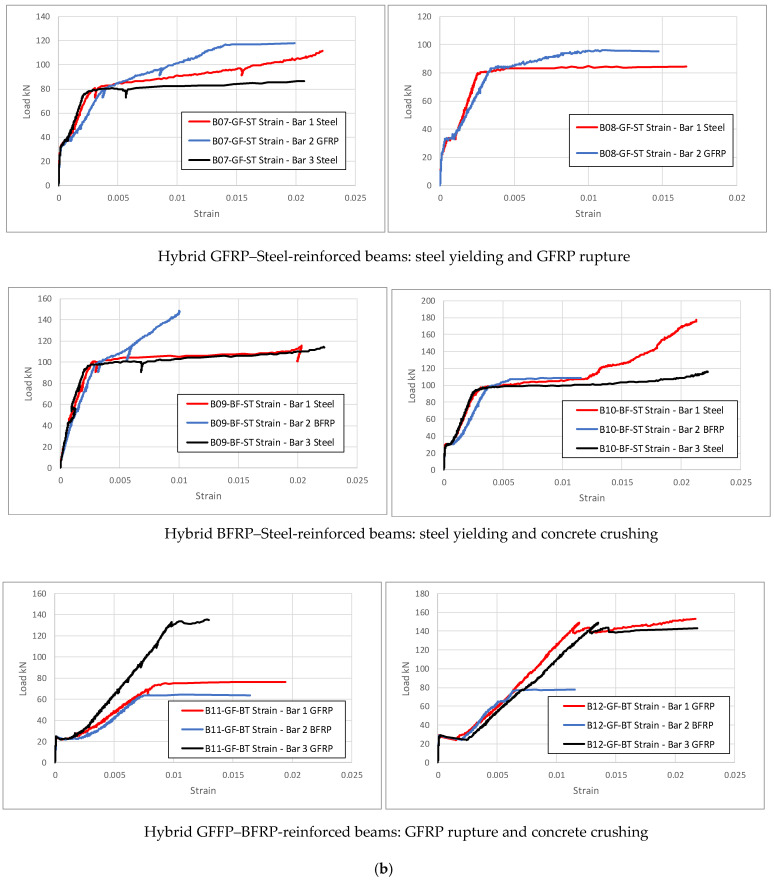
(**a**) Reinforcement tensile strain measurements for all the tested beams. (**b**) Reinforcement tensile strain measurements for all the tested beams.

The tensile strains in the steel, GFRP, and BFRP reinforcement bars recorded during testing of all the beams are plotted in Figure 11a,b. Steel yielding and GFRP rupture or BFRP debonding are evident in the strain measurement and agree well with the failure modes observed during the tests and predicted from the analytical investigation.

### 4.4. Investigation Outcomes

The results of the analytical and experimental investigations reveal the following outcomes:

1. Load Capacity: GFRP- and BFRP-reinforced beams generally exhibited higher ultimate load capacity compared to steel-reinforced beams; 8% and 12%, respectively. Hybrid GFRP–Steel and BFRP–Steel-reinforced beams also show a higher load capacity compared to steel-reinforced beams; 8% and 17%, respectively. Hybrid GFRP–BFRP-reinforced beams do not improve the load capacity due to rupture of the BFRP bars.
2. Deflection: GFRP- and BFRP-reinforced beams show significantly lower deflection at failure compared to steel-reinforced beams (62% and 42% of the steel-reinforced beams, respectively). Hybrid GFRP–Steel and BFRP–Steel-reinforced beams improve this behavior, where they show 90% and 48% of the steel-reinforced beams’ deflection.
3. Failure Modes: GFRP-reinforced beams failed due to GFRP rupture, while BFRP beams experienced debonding or concrete crushing before rupture. Hybrid GFRP–Steel and BFRP–Steel-reinforced beams first exhibited steel yielding, followed by GFRP rupture for GFRP-reinforced beams or concrete crushing for BFRP-reinforced beams. All failure modes agree with those predicted analytically.
4. Ductility: GFRP- and BFRP-reinforced beams exhibited lower ductility compared to steel-reinforced beams, as evidenced by their ductility indices; the ductility indices of steel-reinforced, GFRP-reinforced, and BFRP-reinforced beams are 7.4, 4.4, and 3.1, respectively. The ductility improves significantly for hybrid systems of GFRP–Steel and BFRP–Steel-reinforced beams; ductility indices are 8.0 and 4.7, respectively.
5. Energy Absorption: The area under the load–deflection curve indicates energy absorption capacity and resistance to failure when subject to repeated or cyclic loading. Steel-reinforced beams show the highest area and a superior behavior to all other reinforced beams. The lowest areas are recorded for the GFRP-reinforced beams and the BFRP-reinforced beams (41% and 23% compared to steel-reinforced beams). The hybrid GFRP–Steel and BFRP–Steel reinforcement systems improve this behavior showing 64% and 41%, respectively, of the area under the load–deflection curve recorded for steel-reinforced beams.
6. Anaytical and Experimental Failure Loads: Table 4 shows some discrepancies between the calculated and experimental failure loads for some beams. These differences can be explained by considering a few key mechanisms. The first is the material property variability, where analytical predictions are based on average values of the mechanical properties, whereas in practice, variability in material quality can affect actual performance. For instance, premature rupture or debonding in BFRP-reinforced beams may reduce the load-carrying capacity relative to the calculated values. The analytical model also assumes ideal bonding between the reinforcement and concrete. However, the experimental results, particularly for BFRP-reinforced beams, show signs of premature debonding, which may reduce the load capacity compared to the prediction. Furthermore, in some hybrid systems, load redistribution between reinforcement types may lead to failure modes that are not fully captured by conventional section analysis. For example, in the GFRP–BFRP hybrid beams, rupture of the BFRP bars occurred before reaching the predicted flexural capacity, thus lowering the actual failure load. Finally, the current ACI 440 guidelines may not fully capture the full behavior, interaction effects, or localized failure mechanisms seen in hybrid or BFRP-only systems. Overall, these discrepancies highlight the complexity of FRP and hybrid reinforcement behavior, reinforcing the need for further experimental validation and refinement of analytical models.

## 5. Conclusions

This study investigates the flexural performance of concrete beams reinforced with GFRP, BFRP, and hybrid systems. This study focuses on understanding how these materials compare to traditional steel reinforcement regarding flexural strength, ductility, cracking behavior, and failure modes. Twelve beams are tested, each group reinforced with one type of material (steel, GFRP, BFRP, or hybrids). A three-point load flexural testing scheme is performed, and deflections, strains, and failure loads are recorded.

Given the limited size of the specimen analyzed in this research, the following general conclusions are drawn from the investigation:

1. Special consideration must be adopted in designing beams reinforced with GFRP or BFRP bars.
2. A longer development length is required when using BFRP bars in reinforcing beams. This is not essentially required for GFRP-reinforced beams.
3. Hybrid GFRP–Steel-reinforced beams showed significantly better ductility and increased toughness compared to GFRP-reinforced beams. Hybrid BFRP–Steel-reinforced beams also showed improved ductility relative to BFRP-reinforced beams, though not exceeding that of steel-only beams.

In general, it can be concluded that GFRP- and BFRP-reinforced beams require careful consideration in design to avoid brittle failure. On the other hand, hybrid GFRP–Steel and BFRP–Steel-reinforced beams may offer a better solution that improves both load-carrying capacity and ductility, especially in critical applications like seismic zones or impact-prone structures.

## Figures and Tables

**Table 1:** Details of the tested beams.

Beam	Bottom Reinf		Top Reinf		Stirrups	Length (m)	Dim (mm)
B01-C B02-C	3 No. 12	Steel	2 No. 10	Steel	No. 8@100 mm	2.0	200 × 400
B03-GF B04-GF	3 No. 12	GFRP	2 No. 10	Steel	No. 8@100 mm	2.0	200 × 400
B05-BF B06-BF	3 No. 12	BFRP	2 No. 10	Steel	No. 8@100 mm	2.0	200 × 400
B07-GF-ST B08-GF-BF	2 No. 12 1 No. 12	Steel GFRP	2 No. 10	Steel	No. 8@100 mm	2.0	200 × 400
B09-BF-ST B10-BF-ST	2 No. 12 1 No. 12	Steel BFRP	2 No. 10	Steel	No. 8@100 mm	2.0	200 × 400
B11-GF-BF B12-GF-BF	2 No. 12 1 No. 12	GFRP BFRP	2 No. 10	Steel	No. 8@100 mm	2.0	200 × 400

**Table 2:** Mechanical properties of concrete, steel, GFRP, and BFRP bars, highlighting key attributes that reflect their respective advantages and limitations in structural performance.

Material	Property	Value
Concrete	28d compressive strength: *f_c_^/^*	25 MPa
	Crushing strains *ε_cu_*	0.003
	Initial elastic modulus *E_c_* = 4700 (*f_c_^/^*)^1/2^	23.5 GPa
	α_1_ and β_1_	0.85, 0.85
Steel bars	Yield stress *F_y-s_*—reinforcement bars Yield stress *F_y-v_*—stirrups	465 MPa 360 MPa
	Elastic modulus *E_s_*	200 GPa
	Yield strain *ε_y-s_*—reinforcement bars Yield strain *ε_y-v_*—stirrups	0.0023 0.0018
GFRP bars	Tensile strength *F_u-GFRP_*	483–690 MPa
	Elastic modulus *E_GFRP_*	35–61 GPa
	Rupture strain *ε_u-GFRP_*	0.01–0.031
BFRP bars	Tensile strength *F_u-BFRP_*	600–1700
	Elastic modulus *E_BFRP_*	50–90 GPa
	Rupture strain *ε_u-BFRP_*	0.018–0.032

**Table 3:** Analytical investigation results.

Beam	Nominal Moment M_n_ (kN·m)	Expected Failure Load P_u-M_ (kN)	Failure Mode Analytically Predicted	Nominal Shear V_n_ (kN)	Expected Failure Load P_u-V_ (kN)
B01-C B02-C	55.5	120	Steel yield—Conc crushing	197	393
B03-GF B04-GF	80.9	175	GFRP rupture—Conc crushing	154	308
B05-BF B06-BF	92.9	201	Conc crushing	157	314
B07-GF-ST B08-GF-ST	62.4	135	Steel yield—GFRP rupture	197	393
B09-BF-ST B10-BF-ST	76.7	166	Steel yield—Conc crushing	197	393
B11-GF-BF B12-GF-BF	92.1	199	GFRP rupture—Conc crushing	197	393

**Table 4:** Experimental investigation results.

Beam	Calc. Failure Load (kN)	Expected Failure Mode	Experimental Failure Load (kN)		Deflection at Failure (mm)		Observed Failure Mode	Capacity Increase%
0B01-C B02-C	120	Steel yield—Conc crushing	152.2 149.5	150.8 ± 1.9	69.8 26.5	48.2 ± 30.6	Steel yield—Conc crushing	--
B03-GF B04-GF	175	GFRP rupture—Conc crushing	156.4 170.6	163.5 ± 10.1	26.6 33.2	29.9 ± 4.7	GFRP rupture—Conc crushing	8%
B05-BF B06-BF	201	Conc crushing	178.2 159.5	168.9 ± 13.2	20.4 19.9	20.2 ± 0.4	BFRP debond—Conc crushing	12%
B07-GF-ST B08-GF-ST	135	Steel yield—GFRP rupture	168.7 156.4	162.6 ± 8.7	36.7 49.5	43.1 ± 9.1	Steel yield—GFRP rupture	8%
B09-BF-ST B10-BF-ST	166	Steel yield—Conc crushing	176.3 177.5	179.9 ± 0.85	22.3 23.6	23.0 ± 0.9	Steel yield—Conc crushing	17%
B11-GF-BF B12-GF-BF	199	GFRP rupture—Conc crushing	135.4 153.7	144.6 ± 12.9	23.8 37.5	30.7 ± 9.7	GFRP rupture—Conc crushing	−4%

**Table 5:** Ductility index and toughness of the tested beams.

Beam	Δ_1_ (mm)		Δ_2_ (mm)		DI = Δ_1_/Δ_2_	A (kN·m)		A/A_control_
0B01-C B02-C	70 26.5	48.3 ± 30.8	8.0 5.1	6.6 ± 2.1	7.4	11.03 3.29	7.2 ± 5.5	1.00
B03-GF B04-GF	26.7 33.1	29.9 ± 4.5	6 7.6	6.8 ± 1.1	4.4	2.52 3.37	2.9 ± 0.6	0.41
B05-BF B06-BF	20.5 20	20.3 ± 0.4	4.5 8.4	6.5 ± 2.8	3.1	2.11 1.22	1.7 ± 0.63	0.23
B07-GF-ST B08-GF-ST	36.8 49.8	43.3 ± 9.2	4.6 6.2	5.4 ± 1.1	8.0	3.29 5.92	4.6 ± 1.9	0.64
B09-BF-ST B10-BF-ST	22.4 23.8	23.1 ± 1.0	3.7 6.2	5.0 ± 1.8	4.7	2.91 3.02	2.9 ± 0.1	0.41
B11-GF-BF B12-GF-BF	23.8 23.7	23.8 ± 0.07	5.1 6.5	5.8 ± 1.0	4.1	2.16 4.38	3.3 ± 1.6	0.45

## Data Availability

The raw data supporting the conclusions of this article will be made available by the authors upon request.
